# Liquid-induced colour change in a beetle: the concept of a photonic cell

**DOI:** 10.1038/srep19322

**Published:** 2016-01-13

**Authors:** Sébastien R. Mouchet, Eloise Van Hooijdonk, Victoria L. Welch, Pierre Louette, Jean-François Colomer, Bao-Lian Su, Olivier Deparis

**Affiliations:** 1 Department of Physics, University of Namur (UNamur), Rue de Bruxelles 61, B-5000 Namur, Belgium; 2 Laboratory of Inorganic Materials Chemistry (CMI), University of Namur (UNamur), Rue de Bruxelles 61, B-5000 Namur, Belgium; 3 Clare Hall College, University of Cambridge, Herschel Road, Cambridge CB3 9AL, United Kingdom

## Abstract

The structural colour of male *Hoplia coerulea* beetles is notable for changing from blue to green upon contact with water. In fact, reversible changes in both colour and fluorescence are induced in this beetle by various liquids, although the mechanism has never been fully explained. Changes enacted by water are much faster than those by ethanol, in spite of ethanol’s more rapid spread across the elytral surface. Moreover, the beetle’s photonic structure is enclosed by a thin scale envelope preventing direct contact with the liquid. Here, we note the presence of sodium, potassium and calcium salts in the scale material that mediate the penetration of liquid through putative micropores. The result leads to the novel concept of a “photonic cell”: namely, a biocompatible photonic structure that is encased by a permeable envelope which mediates liquid-induced colour changes in that photonic structure. Engineered photonic cells dispersed in culture media could revolutionize the monitoring of cell-metabolism.

The male *Hoplia coerulea* (Fig. 1a) is a blue-violet beetle from the family Scarabaeidae that lives in South of France and North of Spain, in sunny places along watercourses and in swamps during the summer. Its blue-violet iridescent coloration is generally explained by light interference within a macroporous photonic structure mostly made of chitin and enclosed by a thin envelope, which is found inside the circular shaped scales covering the beetle elytra and thorax^1^ (Fig. 1b–d). So far, the thin envelope has not attracted much attention because it is thought not to affect the coloration due to its subwavelength thickness of about 100 nm. However, scientists discovered reversible water-induced colour changes in the beetle elytra accidentally, after a dead specimen was placed in a wet glass vessel during a field collection^2^, noting that the blue-violet coloration turned green upon contact with water^2^,^3^,^4^. This hygrochromic behaviour was previously explained as resulting from the photonic structure being filled by water^2^. Nevertheless, whether this hygrochromic behaviour is only related to the filling of liquid into the photonic structure of the scale is questionable. Taking this question on board, various liquids of different molecule sizes, functional groups and polarities are investigated here (water, ethanol, methanol, isopropanol are among those tested). Moreover, an intriguing phenomenon is revealed. When water and ethanol are compared, these optical changes appear to be much faster with water than with ethanol in spite of the facts that (1) water forms a high static contact angle on the scales whereas ethanol spreads immediately, without forming a stable droplet and (2) the scale envelope forms a barrier for liquid penetration to the photonic structure.

Recently, part of the development of bioinspired materials has come to rely upon natural porous materials found in living organisms exhibiting properties such as structural coloration^6^,^7^, hygrochromism^2^,^5^,^8^,^9^, fluorescence^10^,^11^,^12^,^13^,^14^,^15^,^16^,^17^,^18^, gas or vapour sensitivity^3^,^19^,^20^,^21^,^22^, thermal management^23^ and/or antireflection^4^,^24^,^25^,^26^,^27^. Examples of this approach include bioinspired smart coatings^28^,^29^. In our study, we characterised colour and fluorescence change dynamics induced by various liquids and analysed in detail the chemical composition of the beetle’s scales. Search for explaining these liquid-induced optical changes led us to develop the novel concept of a “photonic cell” (Fig. 1e), an analogy with a biological cell of which the (selectively permeable cell-) membrane controls the exchanges with its environment. The concept of photonic cell could be revolutionary. For instance, the metabolism of cells could be monitored using biocompatible, optically changing flakes of engineered photonic cells, dispersed in culture media. Microtexture control and chemical functionalisation of the envelope could be used to engineer the photonic cells, in order to detect visually the release or consumption of substances in the culture medium. The easy removal of the photonic cells from the culture medium is a clear advantage in this context.

## Results

### Liquid-induced colour and fluorescence changes

**Figure 1:**
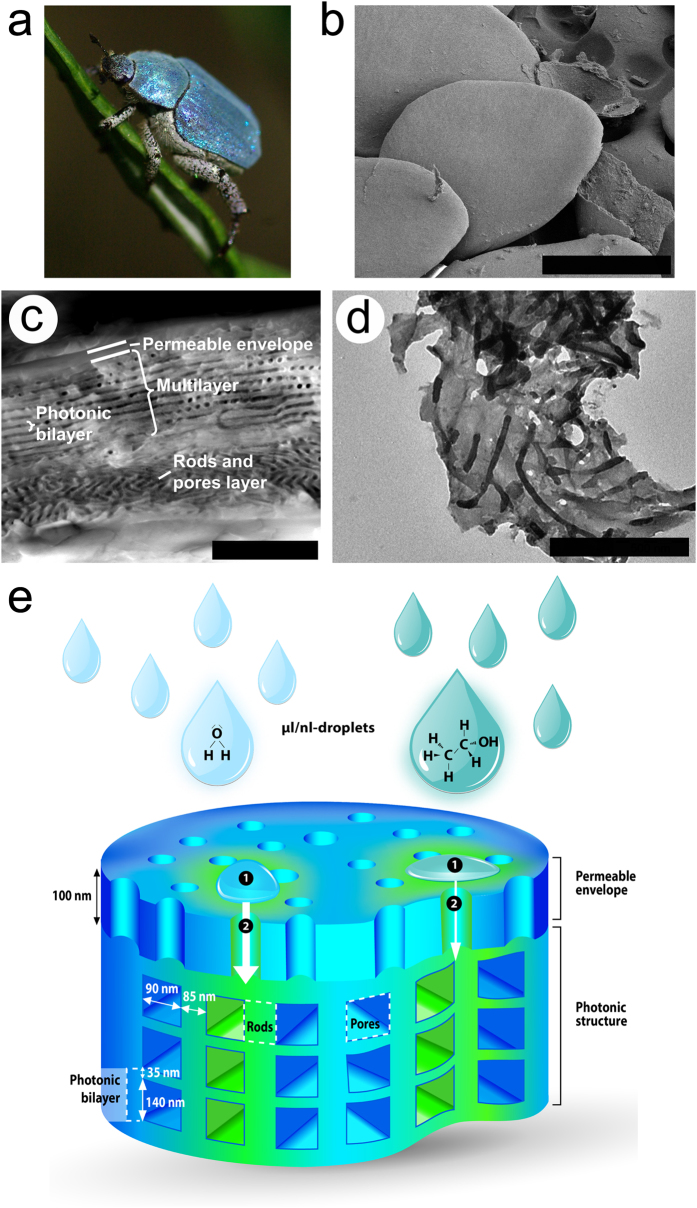
“Photonic cell” of male *H. coerulea* beetle. (**a**) Photograph of male *H. coerulea* beetle. (**b**) Scanning electron microscopy (SEM) image of scales covering the beetle elytra. Scale bar: 40 μm. (**c**) SEM image of a scale cross section. The porous multilayer, enclosed by a thin envelope, gives rise to blue-violet coloration. Scale bar: 2 μm. (**d**) Transmission electron microscopy (TEM) image of mixed air-cuticle layer. Scale bar: 1 μm. (**e**) In spite of the fact that water droplets form a larger contact angle (1) on the beetle scales than ethanol droplets, the permeability of the envelope (2) to water is higher than to ethanol certainly due to the physicochemical properties of the envelope.

**Figure 2:**
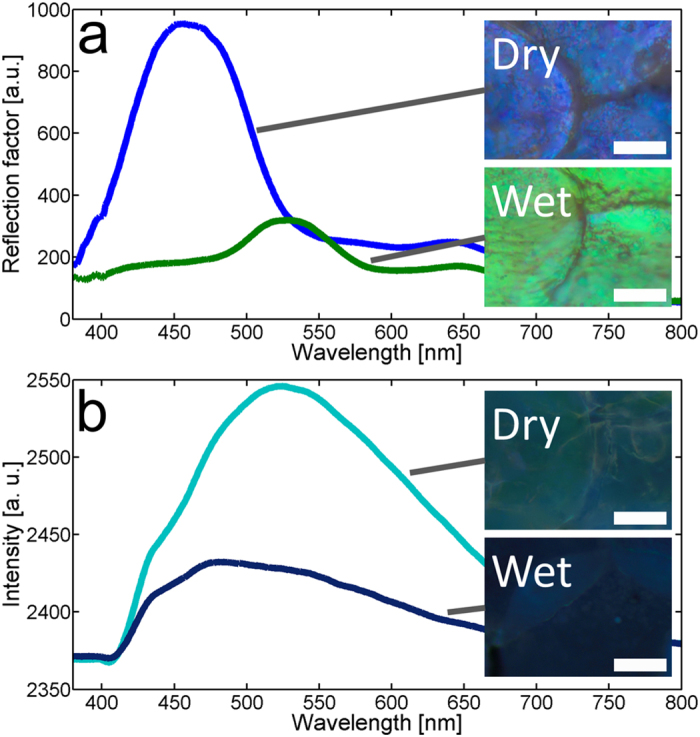
Optical changes induced by contact with water in the scales of the male *H. coerulea* beetle. (**a**) The colour (reflected light), when illuminated by a white lamp, turns from blue to green. (**b**) Fluorescence emission from the scales turns from greenish-blue in the dry state to a dark blue colour in the wet state. Scale bars: 20 μm.

The beetle scales have an average diameter of about 80 μm (Fig. 1b) and an average thickness of about 3.5 μm. The porous multilayer within each consists of a periodic stack of a dozen bilayers and is enclosed by a thin envelope whose thickness is about 100 nm^1^,^2^. Each bilayer (thickness ca. 175 nm) is made of a dense cuticle material layer and a porous spacer layer, the latter being actually a set of rods separated by air voids (Fig. 1c). The rod orientations vary within one single layer as well as from one layer to another. However, these orientation variations as well as the small size of the rods do not result in any optical effect. The envelope, an extra layer covering the dozen bilayers, has a negligible effect on the observed coloration because of its subwavelength thickness. The colour change induced by soaking in water has previously been explained as the filling of the air pores (*n*_air_ = 1.00) of the chitin structure (*n*_chitin_ = 1.56^30^) by water (*n*_water_ = 1.335^31^), giving rise to an increase in effective refractive index (RI) in the porous layers^2^. On the other hand, we found fluorescence emission from the beetle scales to be modified upon wetting. Indeed, when illuminated by UV light, the greenish-blue fluorescent colour exhibited by the sample in the dry state turns to dark blue upon wetting (Fig. 2). Since the photonic structure controls the fluorescence emission^13^, the fluorescent emission is modified as a result of the RI increase.

**Figure 3:**
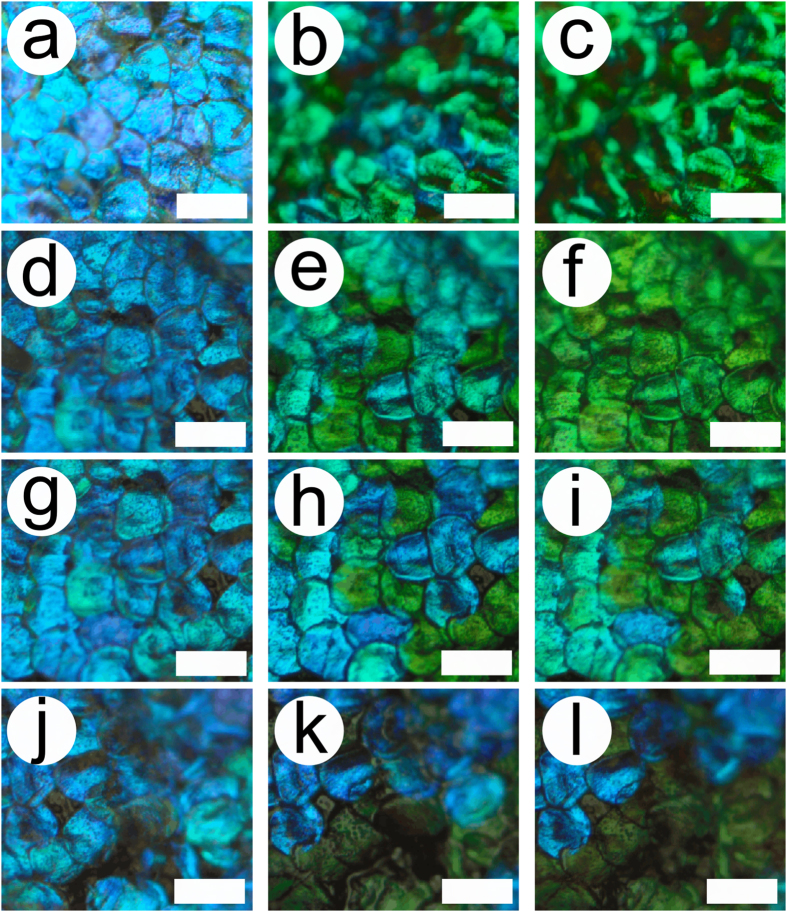
Colour changes induced by liquid droplets. Scales of the male *H. coerulea* beetle turn green after the deposition of 0.5 μl droplets: distilled water (**a**–**c**), absolute ethanol (**d**–**f**), propanone (**g**–**i**) and methylbenzene (**j**–**l**). Ambient state (**a**,**d**,**g**,**j**); ongoing colour change (**b**,**e**,**h**,**k**) at mid-time between ambient and wet states; wet state (**c**,**f**,**i**,**l**). In the case of methylbenzene, the darker green coloration is due to its higher refractive index (RI). In (**i**) and (**l**), not all scales are green, due to evaporation of the liquid. Scale bars: 100 μm.

Colour changes in the beetle elytra were followed in real time by optical microscopy, using 0.5 μl droplets of various liquids (distilled water, methanol, absolute ethanol, 2-propanol, propanone, acetonitrile, methylbenzene and ethoxyethane). A selection of video frames (Supplementary Movies 1 and 2) is presented in Fig. 3. The liquids were chosen according to their chemical properties (Supplementary Table 1) - specifically molecular functional groups (alcohol, ketone, nitrile, ether…), dipole moments (methylbenzene is non-polar whilst the others are polar), molecule sizes (methanol, ethanol and 2-propanol). For each liquid tested, a reversible colour change took place following a similar scenario: some scales instantly turned to green as soon as the droplet was deposited; the other scales turned gradually, one by one, from blue-violet to green, at different speeds. After a while, almost every scale in contact with the liquid was green (Fig. 3). For 2-propanol, propanone, acetonitrile, methylbenzene and ethoxyethane, liquid evaporation was faster than the colour change process; some scales did, however, turn green before the liquid totally evaporated. With water, all scales observed with optical microscopy videos turned green almost immediately (within less than 1 s), whereas colour changes started later with ethanol. With methylbenzene, the observed green colour was darker than with other liquids (Fig. 3j–l). This was due to the RI of liquid methylbenzene (Supplementary Table 1) which is higher than that of the other liquids, leading to a lower RI contrast with respect to chitin.

This observation is radically different from the case of e.g., *Morpho* wings. In that case, it is well known that when a droplet of ethanol is deposited on one wing, it tends to fill the pores of the open photonic structure, whereas, when a droplet of water is deposited, the water does not enter the structure, since Morpho wings are superhydrophobic^7^.

**Figure 4:**
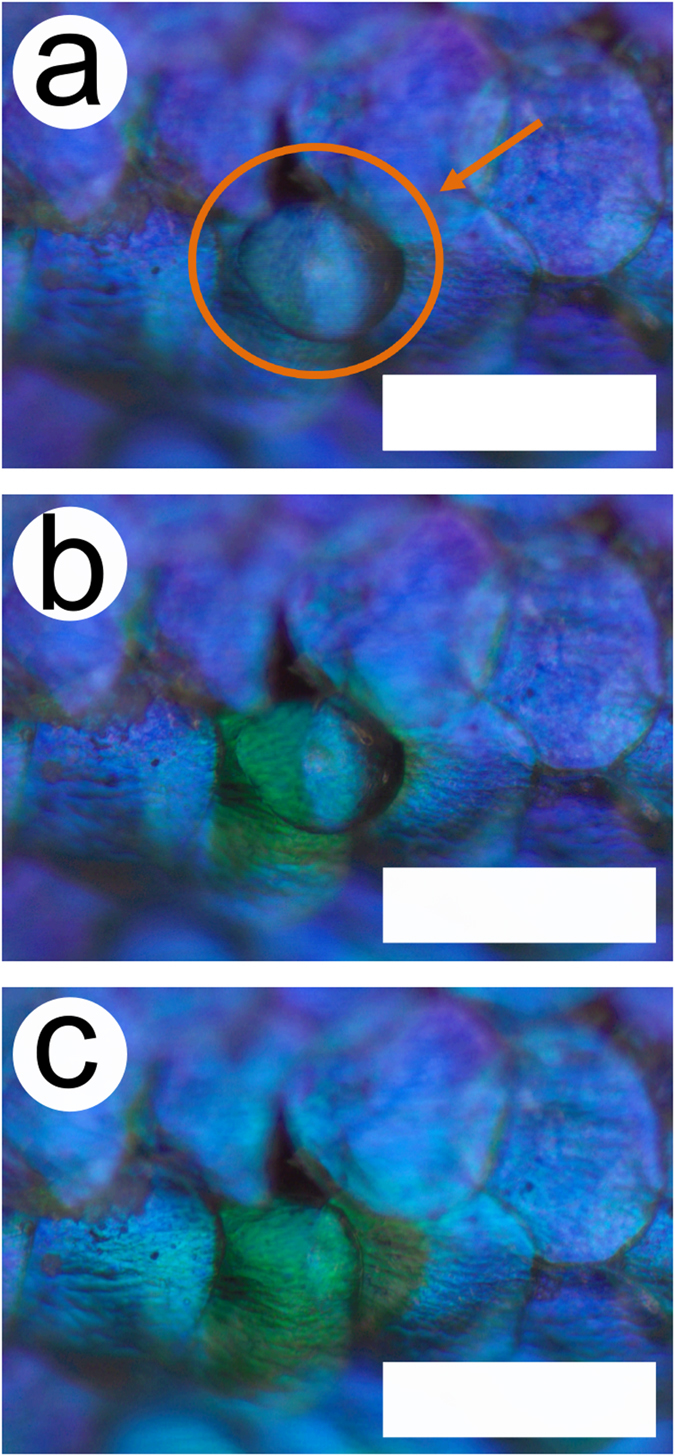
Colour change induced by a nanodroplet. Scales of the male *H. coerulea* beetle turn green after the deposition of nanodroplets from a commercial spray. Just after deposition - orange arrow and circle (**a**), when the scale starts to turn to green (**b**) and when all of the area near the droplet location is green (**c**). Scale bars: 100 μm.

Additionally, nanodroplets of about 1 nl were sprayed on the beetle cuticle, using a commercial water spray (Fig. 4 and Supplementary Movie 3). The size of nanodroplets (radius of about 40 μm) was similar to the size of the scales. From these videos, it becomes obvious that the colour change merely occurs at the place where the nanodroplet is deposited, i.e., on the top side of the scales, thereby excluding the likelihood of liquid penetration from the bottom side or from the peduncle that attaches the scales to the elytra.

For a quantitative evaluation of colour changes, reflection factor spectra (i.e., reflected intensity normalised to a standard white diffusor) of beetle elytra were measured in real time using a fibre-optic microspectrophotometer following the deposition of 0.5 μl droplets. Depending on the liquid, different colour change dynamics were recorded (Supplementary Fig. 1). In ambient conditions, the reflection peak is located at 472 ± 8 nm with a full width at half maximum of 141 ± 19 nm at normal incidence, which is consistent with previous reports^1^,^2^. After the droplet deposition, the peak shifts to about 530 nm for water and to about 540 nm for ethanol or methanol. Upon drying, the initial colour is recovered. The measured peak shifts are consistent with ref. 2.

**Figure 5:**
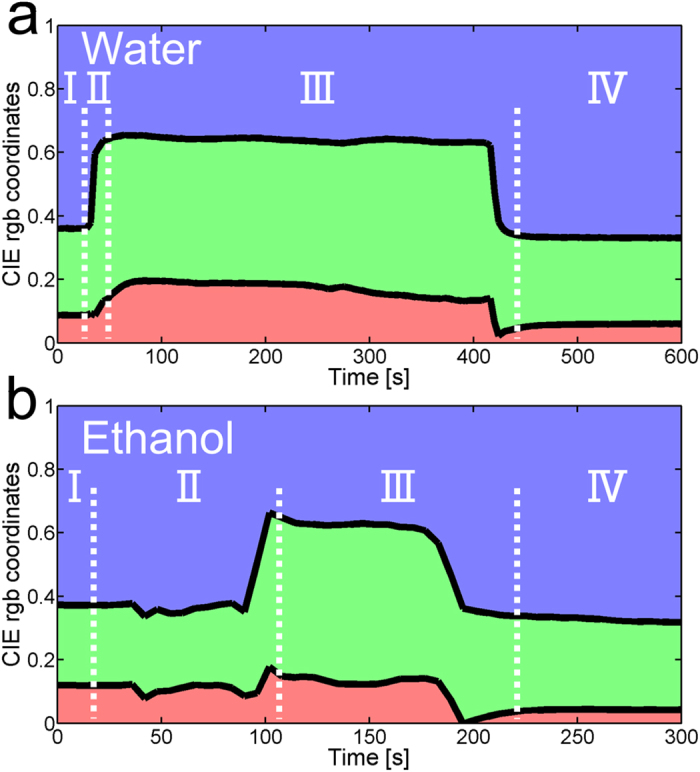
Liquid-induced changes of CIE *r*, *g* and *b* coordinates. Following the deposition of water (**a**) and ethanol (**b**) droplets on *H. coerulea* elytra, the CIE coordinates are modified. Before the droplet deposition (time slot I), the colour is predominantly blue. When the droplet is deposited (at the beginning of time slot II), the colour changes from blue to green. The droplet then evaporates at ambient temperatures (time slot III). When the scales are dried (time slot IV), the initial colour is recovered. Paradoxically, the elytra turn to green earlier and faster with water than with ethanol.

The normalised CIE red (*r*), green (*g*) and blue (*b*) coordinates were calculated from the reflected intensity spectra and plotted as function of time following the droplet deposition (Fig. 5). Offset time (*t*_offset_), rise time (*t*_rise_) and fall time (*t*_fall_) related to colour change dynamics were determined from the changes of *g* coordinate (Supplementary Table 2). They correspond, respectively, to the time taken for this coordinate to rise from the baseline level to 10% of its maximal value *g*_max_, to increase from 10% to 90% of *g*_max_ and to decrease from 90% to 10%. The offset and rise times were found to be shorter for water (*t*_offset_ = 4 s and *t*_rise_ = 3 s) than for ethanol (*t*_offset_ = 61 s and *t*_rise_ = 11 s). In other words, the appearance of green colour starts earlier and is faster with water (Fig. 5a) than with ethanol (Fig. 5b). The *b* coordinate undergoes complementary change to the *g* coordinate as the colour changed from blue to green (Fig. 5). A comparison between the three tested alcohols is presented in Supplementary Fig. 2. For methanol (smallest tested alcohol molecule), the colour change starts earlier (*t*_offset_ = 7 s) and is faster (*t*_rise_ = 10 s) than for ethanol. In the case of 2-propanol (largest tested alcohol molecule), the *g* coordinate becomes higher than the *b* coordinate, but not as high as with water and with the other alcohols. This behaviour confirms what was observed by optical microscopy: some scales turned green, but not all of them (Fig. 3). For 2-propanol, as compared with ethanol, we note that the offset time (*t*_offset_ = 32 s) is shorter, whilst the rise time (*t*_rise_ = 19 s) is longer. The *g* coordinate maximal value is lower, implying a less striking colour change. Methanol, ethanol and 2-propanol have the same molecular functional group (i.e., alcohol) and therefore similar chemical properties. However, they differ in their molecular size and this could explain why the colour changes appear to depend upon the alcohol species. Propanone also gives rise to colour change, with an offset time *t*_offset_ = 24 s and rise time *t*_rise_ = 5 s. For the three other liquids (acetonitrile, methylbenzene and ethoxyethane), the *g* coordinate increases, without becoming higher than the *b* coordinate, corresponding to a weaker colour change.

### Contact angle measurements

The elytra of *H. coerulea* appear here to be hydrophilic, whereas some insect wings, especially butterfly wings, are known to be hydrophobic^7^. In order to gain insight into the wettability of the various tested liquids, we measured the static contact angle *θ*_m_ formed by a liquid droplet deposited on the insect cuticle, which is not a flat surface (Supplementary Table 1). In the case of water, the measured contact angle was significantly smaller (76 °) than the one evaluated for a flat chitin surface (102 °). We note that the scales form a corrugation on the elytron surface, which could, in principle, influence the wettability, generally increasing the contact angle^32^. For some liquids (methanol, ethanol, 2-propanol, propanone, methylbenzene and ethoxyethane), the droplets spread on the surface with the consequence that the contact angle could not be measured. This means that the beetle scales have a very high affinity for the liquids, in these cases. This spreading is confirmed by Young’s equation, in which cos *θ* is larger than 1, meaning that these liquids spread on the surface without forming a stable droplet^32^. On the other hand, water and acetonitrile, whose theoretical contact angles can be calculated, give rise to measurable contact angles (Supplementary Table 1). Comparing droplets of water and ethanol, the ethanol droplet is found to spread on the surface, while water is stable and forms a measurable contact angle (Supplementary Table 1). This observation may seem counterintuitive, since the colour change occurs more rapidly with water than ethanol and is discussed below.

### Material chemical composition

The contact angle of water on the beetle scales is measured to be about 76° (i.e., hydrophilic surface) while the theoretical value on a pure chitin surface is found to be equal to 102° by our calculation and was evaluated at 105° by ref. 33. The chemical composition of the beetle scale material is a key factor in understanding the hydrophilicity of the envelope. Indeed, the insect cuticle is not only composed of chitin, but also of other extracellular macromolecules like proteins, lipids, phospholipids, wax, etc^6^ composed of esters, ketones, aldehydes, etc^34^. Chitin ((C_8_H_13_O_5_N)_n_) is a biopolymer resulting from the copolymerisation of at least 50% of *N*-Acetyl-*β*-glucosamine and *D*-glucosamine. We investigated the chemical composition of the beetle elytra using X-ray photoelectron spectroscopy (XPS) (Supplementary Figs 3 and 4). The main XPS peaks are those of carbon, oxygen and nitrogen (Supplementary Table 3), as might be expected. The beetle elytron surface is composed of 83.64% of carbon and 9.90% of oxygen. This is consistent with a surface made of polysaccharide chains such as chitin and with the literature^34^,^35^. Chlorine, phosphorus, sodium and potassium are found in very small amounts. Calcium was also detected in some measurements. To our knownledge, chlorine and potassium have never been detected in insect wings but have been found in beetle mandibles^36^. Termite mandibles also contain chlorine^37^. The presence of sodium and phosphorus on beetle elytra has been however reported in the literature^8^,^34^. Depth profiles (performed using clusters of Ar^ + ^ions) show that these atom contents are also present across the samples, irrespective of depth.

The origin of Cl, P, Na, K and Ca in the cuticle is less clear. Phosphorus could be due to proteins or phospholipids, where it seems to be linked to oxygen (P-O), according to Gaussian-Lorentzian fits of XPS measurements. It appears that mixtures of NaCl, KCl and CaCl_2_ are present in the cuticle. The Cl atom content is furthermore almost equal to the sum of Na, K and Ca atom contents. They would appear to induce the hydrophilicity of the elytron surface since hydrophobic polymer substrates are known to become hydrophilic when they are doped by ions^38^,^39^,^40^. Dissolution of these salts into the liquids likely explains their role in the penetration of the liquids into the scales. Indeed, NaCl, KCl and CaCl_2_ are very common natural salts, which are very soluble in water and less so in other solvents like ethanol^41^. They are present in the cells of living organisms. The presence of these five elements (Cl, P, Na, K and Ca) due to exchange with intracellular chemical entities during the building of the scale is plausible, not least since Ghiradella showed that diffraction gratings found in iridescent scales of lycaenid butterflies are shaped by the smooth endoplasmic reticulum of their scale cells^42^. Of course, whether the endoplasmic reticulum is involved in other kinds of scale building process and whether such a transfer of elements from the cell to the scale cuticle takes place is not known.

## Discussion

The challenging question here is to understand how, at the microscopic level, liquids penetrate through the envelope into the scale and fill the pores of the photonic structure. As mentioned above, the envelope does not affect the observed coloration, but acts as a barrier that delays the penetration of liquids. The physicochemical properties of liquids and cuticle material play a key role in the observed colour change dynamics. Among the scales that changed colour immediately, we noted that some of them were already damaged (i.e., broken envelope) before having any contact with liquids. We postulate that each scale turning instantly from blue-violet to green was damaged. As a matter of fact, the male *H. coerulea* beetle displays a remarkable and puzzling example of colour change induced by contact with liquids, in spite of the fact that the porous photonic structure is not open to the environment, but rather is enclosed by an envelope. The underlying function of liquid-induced colour change of this insect has not been understood so far. The combination of warm and wet conditions encountered by the beetle (in wet sunny places) could induce such colour changes in the natural environment, possibly playing a role in camouflage or thermoregulation.

Chitin, a common biopolymer, has been extensively studied for a long time. The chitin forming the respiratory system and the gut of insects is known to be permeable to water^43^,^44^, likewise, the carapace of fiddler crabs *Uca*^45^ and as well as the carapace and gills of horseshoe crabs *Limulus polyphemus*, with the latter differing from the former by having no calcaneous component^46^. In 1935, Yonge^47^ highlighted the fact that chitin from the foregut of lobster *Homanus vulgaris* was more permeable to water than to alcohols. He postulated that alcohols were not able to pass freely through a chitin membrane because they might dehydrate chitin. In 1984, water and ethanol fluxes through a chitin membrane were quantified at atmospheric pressure. Water flux appeared to be 100–300 times greater than ethanol flux^48^. The much larger water flux was tentatively explained by the smaller size of water molecules, some aspects of their physicochemical properties and the fact that water molecules could pass across the chitin membrane through different routes than ethanol. Of course, other subtances in the scale envelope, such as lipids, may influence its permeability to liquids. Lipids have been shown to play just such a role in the keratin layers of snake skin^49^. All these early observations tend to support our model, according to which water penetrates faster through the envelope than ethanol, thereby explaining the previously unexplained colour change dynamics observed in *H. coerulea*.

One possible explanation for the observed liquid-penetration into the scales could be that their envelopes were porous. SEM and TEM observations (Fig. 1b–d) evidenced the absence of both macropores (i.e., pore diameter higher than 50 nm) and mesopores (i.e., pore diameter between 2 and 50 nm) in the envelopes. Micropores (i.e., pore diameter smaller than 2 nm) are not observable by these techniques. High-Resolution TEM observations as well as nitrogen physisorption porosimetry did not allow us to reveal the existence of micropores because of the degradation of the samples by the electron beam and because of the very small volume of pores in the material, respectively. However micropores (at least larger than methylbenzene molecule size, i.e., 0.6 nm) could be present in the envelope though not observable by these techniques.

The mechanism at the origin of the colour change dynamics observed in *H. coerulea* beetle is summarised in Fig. 1e. In spite of the fact that water droplets form a large contact angle on the scales whereas ethanol spreads on them, water molecules penetrate much more easily through the envelope because of the higher permeability of chitin to water than to ethanol. The latter property is related to both the microporosity of chitin and its chemical composition. Our XPS study revealed the presence of salts (NaCl, KCl and CaCl_2_) in the scales’ cuticle material that produces the hydrophilicity of the surface. Since these salts are very much soluble in water but only slightly soluble in alcohol, they most likely foster the capillary flow of water into the micropores. The molecule size influences the liquid penetration through the micropores. Actually, the envelope acts as a sieve allowing smaller molecules to penetrate more easily. Furthermore, the longer the molecule, the lower the dipole moment (Supplementary Table 1), and consequently there is a sizeable reduction in permeability of different alcohols. The optical system of the scales, i.e., an internal photonic structure enclosed by an envelope that acts as a permeable barrier controlling the exchange with the outer environment, is reminiscent of a biological cell, of which the vital functionalities are protected by a membrane mediating the exchange with the outside world. By analogy, we term the beetle optical system a “photonic cell”. This new concept has great potential for the development of novel smart materials using a bioinspiration approach.

In summary therefore, water and other liquids induce reversible changes of optical properties in *H. coerulea* beetle scales: the colour turns from blue-violet to green, whereas the fluorescence turns from greenish-blue to dark blue. The dynamics of these changes depend on the liquid type e.g., being much faster with water than ethanol. Both colour and fluorescence changes are due to rapid liquid filling of the internal macroporous photonic structure through its permeable envelope. The dynamics can be explained by the identification of salts in the cuticle material which enhance its permeability to water.

The design of the optical system of the scales is that of a photonic structure enclosed in a functionalised membrane (the envelope) that acts as a semi-permeable barrier to the outside. In other words, it is analogous to a biological cell, in which the membrane mediates exchange with the environment while the internal structures perform the cellular functions. Thus, our analysis suggests that we might style this system as a “photonic cell”, i.e. a photonic structure enclosed in a permeable (and putatively regulating) membrane. This insight may pave the way to the development of a new category of smart photonic materials.

The applications of our photonic cell concept go far beyond the sensing of volatile substances as it may be deduced from our results. One may envisage paints which contain photonic cell flakes that are functionalised in order to selectively detect specific volatile substances. Another family of applications could be sensing of biochemical products in culture media. The production of the substances could be detected by photonic cell flakes that were surface-functionalised in order to be permeable only to these substances.

## Methods

### Optical measurements

A 0.5 μl droplet of liquid (distilled water, methanol, absolute ethanol, 2-propanol, propanone, acetonitrile, methylbenzene or ethoxyethane) was deposited on a dead beetle elytron using a micropipette. Real-time optical microscope observations were performed with an Olympus BX61 microscope, combined with an Olympus XC50 camera and an Olympus BX-UCB light source. One video frame enabled about 200 scales to be observed. The reflection factor (reflected light intensity normalised to the intensity reflected by an Avantes WS-2 white diffusor and corrected for noise), was also recorded in real time following droplet deposition with an Ocean Optics QE65 Pro spectrophotometer connected to the microscope. Fluorescence emission spectra were measured with the same microscope and spectrophotometer as used for the reflection changes, as well as with a Lumen Dynamics X-cite Series 120PCQ UV-lamp. The size of the analysed spot on the elytron extended over about several hundreds square micrometres, thus only a few scales were examined in each case. The measurements were performed at zero incidence and detection angles. From the reflection factor spectra, CIE 1931 coordinates (*X*, *Y*, *Z*) were calculated^50^,^51^,^52^. Applying a conversion matrix to these coordinates, the CIE RGB coordinates were derived, normalised such that *r* = *R/*(*R* + *G* + *B*), *g* = *G/*(*R* + *G* + *B*) and *b* = *B/* (*R* + *G* + *B*) and plotted as function of time. The offset time (*t*_offset_), rise time (*t*_rise_) and fall time (*t*_fall_) of the colour change were defined, respectively, as the time taken for the *g* coordinate to rise from the baseline level to 10% of its maximal value, to increase from 10% to 90% of the maximal value and to decrease from 90% to 10% of the maximal value. Nanodroplets, whose sizes (radius of about 40 μm) were similar to scales of the beetle elytra, were also sprayed on the beetle elytra (spray bought in a drugstore) at a distance of about 20 cm, while the elytra were observed with real-time optical microscopy.

### Photonic structure morphology

Scanning Electron Microscope (SEM) analysis of the scale morphology was performed with a Fei Nova Nanolab 200 Dual-Beam microscope and a Fei Quanta 200 F microscope. We observed both sides of intact scales and cross sections of fractured scales. The elytra were cut using a scalpel and attached to the sample mount with adhesive tape. A Fei Tecnai 10 microscope was used for Transmission Electron Microscope (TEM) observations. Scales were scraped off the beetle elytron, ground in a mortar and deposited on copper grids with an ethanol solution.

### Surface chemical composition

In order to determine the chemical composition of the beetle elytra, X-ray Photoelectron spectroscopy (XPS) analysis was performed using a Thermo Scientific Escalab 250Xi electron spectrometer with 50 W Al-K*α* radation (*E* = 1486.6 eV), giving rise to a 200 μm spot size. In order to avoid surface charging, samples were flooded with low energy electrons during the analysis, using a flood gun. Elements present on the elytron surface were determined by a set of three analyses performed at three different spots. For each spot, a broad-band spectrum from (0 to 1350 eV) was measured with an energy resolution of 1.16 eV and a pass energy of 150 eV. The atomic content (in %), assessed on the basis of the peak area, was averaged between the results of the three spots. High-resolution spectra were measured within 20 eV intervals across the C1s, O1s, N1s, Cl2p, P2p, Na1s and Ca2p peaks and fitted with Gaussian-Lorentzian components, thanks to Thermo Scientific Avantage Data System software. The K2p peak was located in the spectrum of C1s and the P2s, in the spectrum of Cl2p. Chemical bonds were determined according to peak energies of the fit components using X-ray Photoelectron Spectroscopy handbooks and databases^53^,^54^,^55^. In order to analyse the chemical composition across the depth of the beetle scale, depth profiles were performed with 4000 eV clusters of Ar^+^ ions (about 1000 ions per cluster) for up to 9.3 h.

### Wettability

The static contact angle *θ* formed by a droplet on a flat chitin surface was calculated for each liquid tested (distilled water, methanol, absolute ethanol, 2-propanol, propanone, acetonitrile, methylbenzene and ethoxyethane) using Young’s equation^56^, 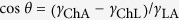, where *γ*_ChA_, *γ*_ChL_ and *γ*_LA_ are the chitin-air, chitin-liquid and liquid-air surface tensions, respectively. First, the values of chitin-liquid surface tension *γ*_ChL_ were derived using 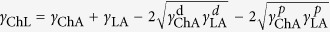
56 with 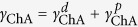 and 
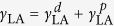
 where *d* and *p* correspond to dispersive and polar interactions. The values of the dispersive and polar components of the chitin-air and liquid-air surface tensions were found in the literature^57^,^58^,^59^,^60^,^61^ for the different liquids.

Static contact angle measurements were performed with a Dataphysics OCA 35 apparatus, at about 22 °C with 0.5 μl and 1 μl droplets of the eight liquids. The reported values *θ*_m_ resulted from averages of at least four measurements.

## Additional Information

**How to cite this article**: Mouchet, S. R. *et al.* Liquid-induced colour change in a beetle: the concept of a photonic cell. *Sci. Rep.* **6**, 19322; doi: 10.1038/srep19322 (2016).

## Supplementary Material

Supplementary Information

Supplementary Movie 1

Supplementary Movie 2

Supplementary Movie 3
