# Supplementary material for: Liquid-induced colour change in a beetle: the concept of a photonic cell
Source: Sci Rep. 2016 Jan 13;6:19322. doi: 10.1038/srep19322 (PMC4725358; doi:10.1038/srep19322)
Supplement: Supplementary Information [file srep19322-s1.doc]

Supplementary Information

Liquid-induced colour change in a beetle: the concept of a photonic cell

Sébastien R. Mouchet, Eloise Van Hooijdonk, Victoria L. Welch, Pierre Louette, Jean-François Colomer, Bao-Lian Su, Olivier Deparis


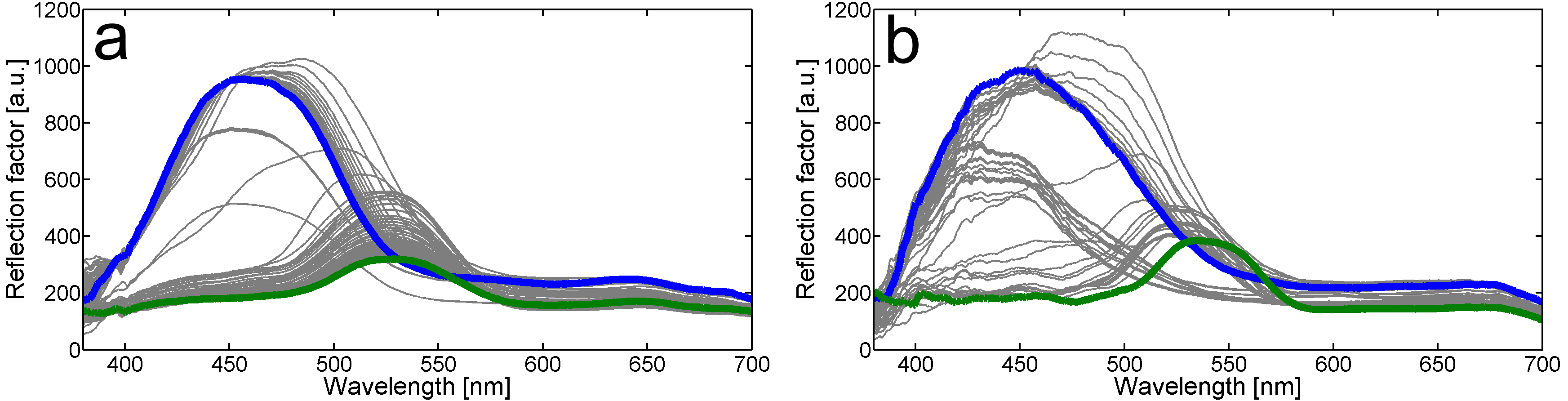


**Supplementary Figure 1 Liquid-induced colour change in beetle scales.** Colour change dynamics of *H. coerulea* elytra induced by the deposition of a water (a) and ethanol (b) droplets. Blue curves: dry state; green curves: wet state; grey curves: intermediate spectra.


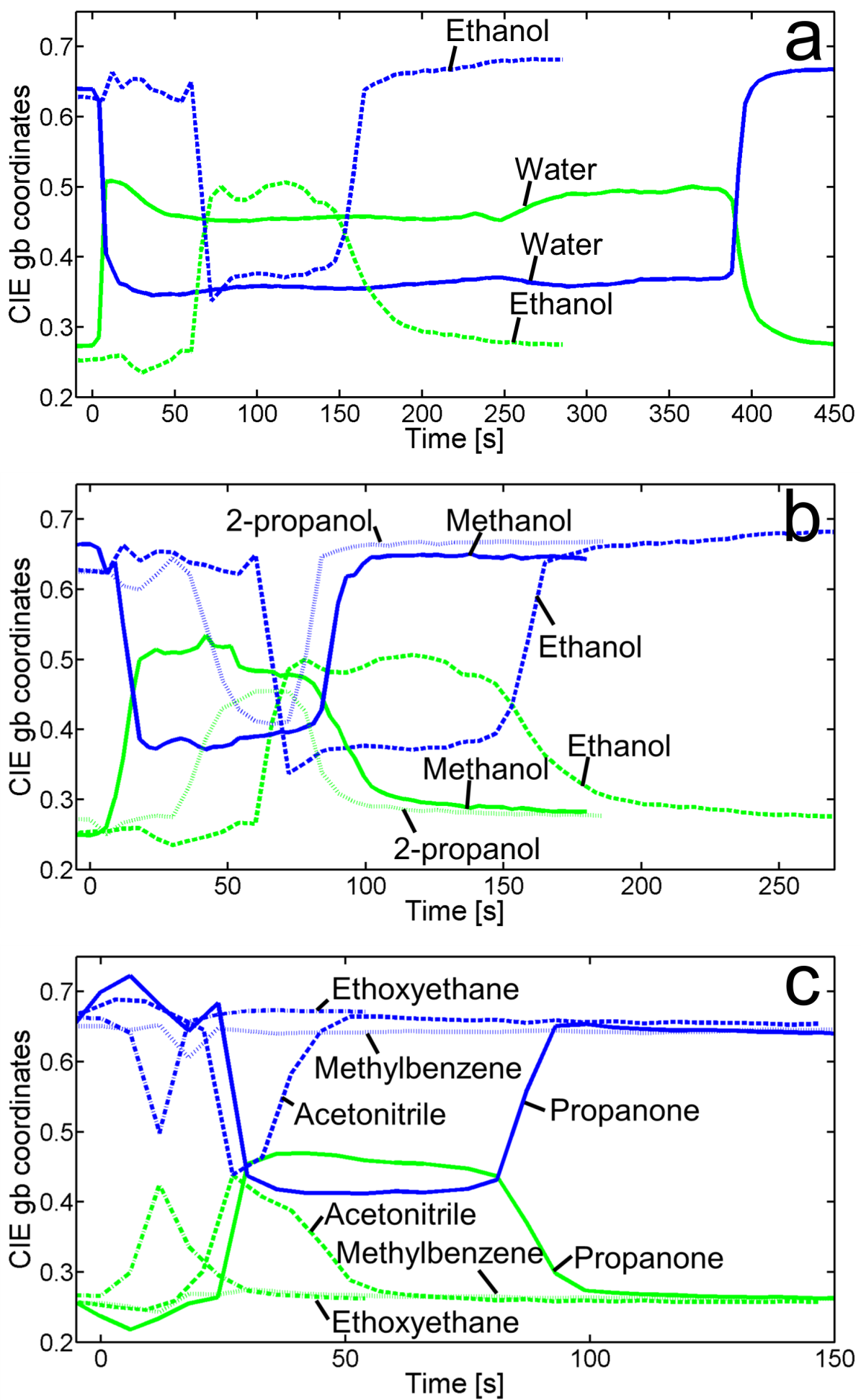


**Supplementary Figure 2** **Changes of CIE *g* and *b* coordinates after deposition of droplets on the beetle elytra.** a) water and ethanol. b) methanol, ethanol and 2-propanol. c) propanone, acetonitrile, methylbenzene and ethoxyethane. *g* and *b* coordinates are green and blue curves, respectively.


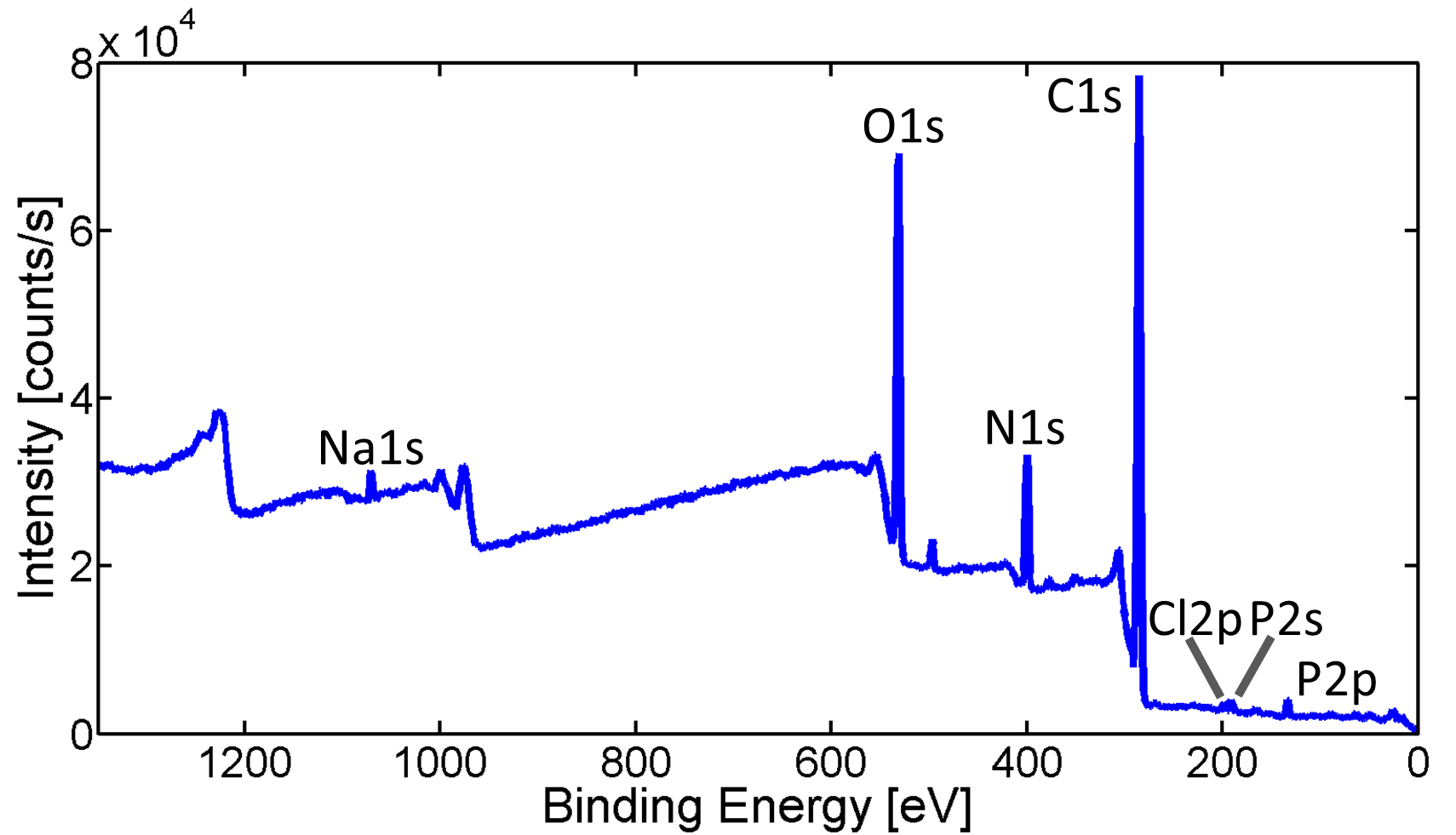


**Supplementary Figure 3 XPS analysis.** Broad-band XPS spectrum measured on one elytron of male *H. coerulea* beetle.


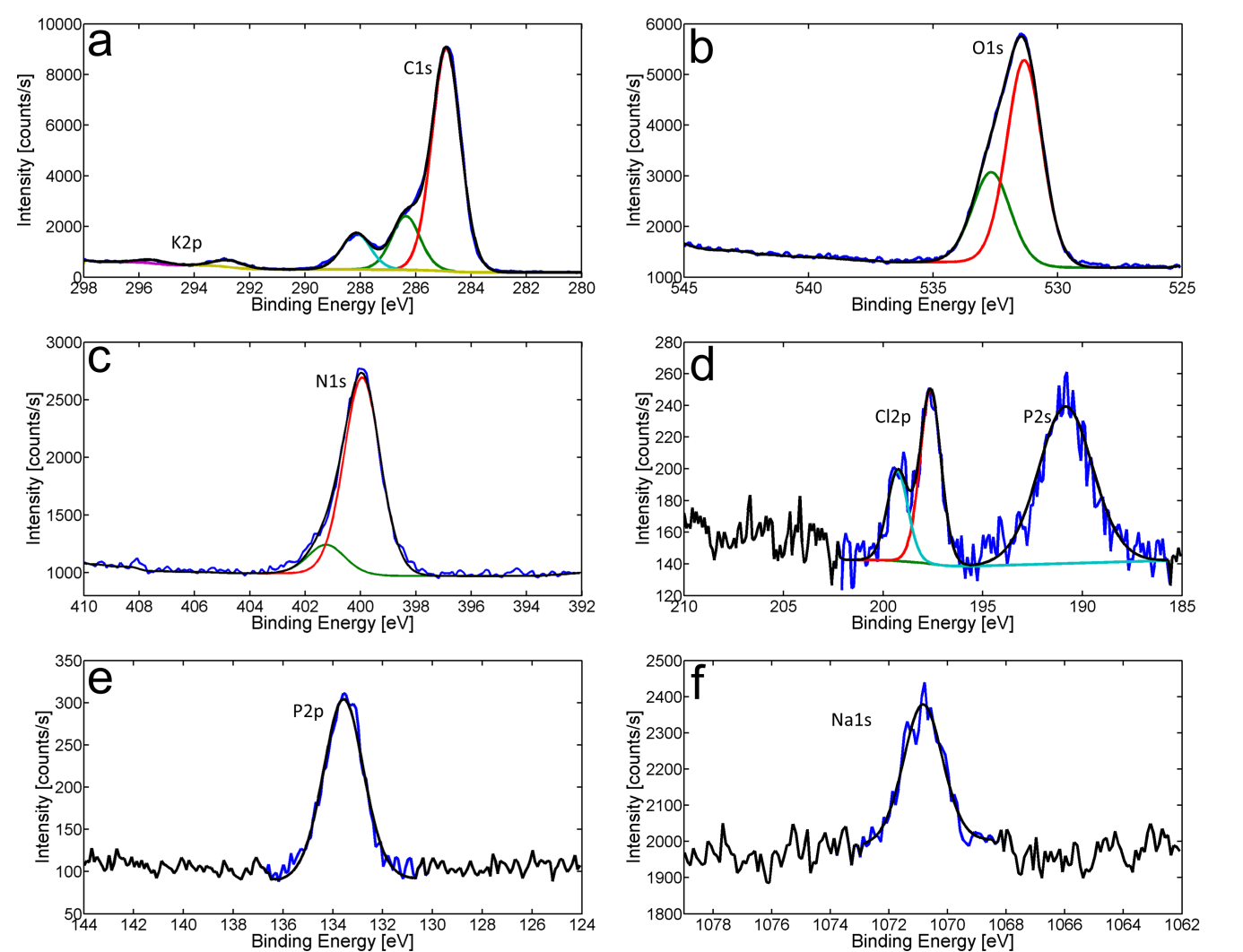


**Supplementary Figure 4** **High-resolution XPS spectra measured on one elytron of male *H. coerulea* beetle.** a) C1s, b) O1s, c) N1s, d) Cl2p, e) P2p and f) Na1s peaks. Spectra were fitted with Gaussian-Lorentzian components.

| Molecule | Dipole moment µ (Debye D)^1^ | Real part of RI ($\lambda=500 \text{nm}$, if nothing else is mentioned) | Chitin-liquid surface tension $\gamma_{\text{LCh}}$ (mN/m) | Contact angle $\theta$ on flat chitin surface (°) | Measured contact angle $\theta_{\text{m}}$ on insect cuticle (°) |
| --- | --- | --- | --- | --- | --- |
| Water  (H_2_O) | 1.854 | 1.335^2^ | 53.1 | 102 | 76±15 |
| Methanol (CH_4_O) | 1.70 | 1.345^3^ | 7.8 | X | X |
| Ethanol (C_2_H_6_O) | 1.69 | 1.365^4^ | 5.8 | X | X |
| 2-propanol (C_3_H_8_O) | 1.58 | 1.375^5^ | 15.5 | X | X |
| Propanone (C_3_H_6_O) | 2.88 | 1.363^4^ | 1.2 | X | X |
| Acetonitrile (CH_3_CN) | 3.924 | 1.344^6^ | 11.7 | 21 | 34±7 |
| Methylbenzene (C_7_H_8_) | 0.375 | 1.501^6^ | 0.6 | X | X |
| Ethoxyethane ((C_2_H_5_)_2_O) | 1.15 | 1.353  ($589.29 \text{nm}$)^7^ | 4.0 | X | X |

**Supplementary Table 1 Physico-chemical properties of the tested liquids.** Dipole moments, refractive index (RI), chitin-liquid surface tensions $\gamma_{\text{LCh}}$, contact angle $\theta$ formed by a liquid droplet on a flat chitin surface and measured contact angle $\theta_{\text{m}}$ formed on the insect cuticle for the eight tested liquids. $\gamma_{\text{LCh}}$ and $\theta_{\text{m}}$ were evaluated thanks to chitin-air $\gamma_{\text{ChA}}$ and liquid-air $\gamma_{\text{LA}}$ surface tensions as well as Young's equation^8^. The value X for $\theta$ means that the calculated value of $\cos\theta$ in Young's equation is larger than 1, consequently the liquid spreads on the chitin surface^9^. The value X for $\theta_{\text{m}}$ means that the droplet is not stable on the surface and flattens immediately after deposition. The experimental error on the water contact angle (15°) is reasonable, if one takes into account the fact that the beetle’s elytra are not flat.

| Molecule | $t_{\text{offset}}$ (s) | $t_{\text{rise}}$ (s) | $t_{\text{fall}}$ (s) |
| --- | --- | --- | --- |
| Water  (H_2_O) | 4 | 3 | 25 |
| Methanol  (CH_4_O) | 7 | 10 | 30 |
| Ethanol  (C_2_H_6_O) | 61 | 11 | 45 |
| 2-propanol  (C_3_H_8_O) | 32 | 19 | 26 |
| Propanone  (C_3_H_6_O) | 24 | 5 | 14 |
| Acetonitrile  (CH_3_CN) | 25 | 10 | 29 |
| Methylbenzene  (C_7_H_8_) | 15 | 6 | 168 |
| Ethoxyethane  ((C_2_H_5_)_2_O) | 3 | 8 | 15 |

**Supplementary Table 2 Offset time $t_{\text{offset}}$, rise time $t_{\text{rise}}$ and fall time $t_{\text{fall}}$ for the eight tested liquids.** Surprisingly, the offset time and rise time are shorter for water than for alcohols, such as ethanol.

| Element | Atom content (%) | Binding Energy Peak (eV) |
| --- | --- | --- |
| C1s | 83.64±6.19 | 284.87±0.12 |
| O1s | 9.90±3.55 | 531.55±0.07 |
| N1s | 4.41±1.70 | 399.94±0.07 |
| Cl2p | 0.67±0.25 | 192.95±3.97 |
| P2p | 0.64±0.48 | 133.31±0.22 |
| Na1s | 0.32±0.23 | 1070.82±0.13 |
| K2p | 0.29±0.24 | 2.92.69±1.84 |

**Supplementary Table 3 Results of XPS measurements on *H. coerulea* elytron surface.** Atom content, biding energy and possible chemical bonds for each detected element.

**Supplementary Movie 1 Colour changes induced by a distilled water droplet.** Scales of the male *H. coerulea* beetle turns to green after the deposition of a 0.5 µl droplet.

**Supplementary Movie 2 Colour changes induced by an ethanol droplet.** Scales of the male *H. coerulea* beetle turns to green after the deposition of a 0.5 µl droplet.

**Supplementary Movie 3 Colour changes induced by a distilled water nanodroplet.** Scales of the male *H. coerulea* beetle turns to green after the deposition of a nanodroplet from a commercial spray.

**Supplementary references**

1. Lide, D. R. *CRC Hanbook of Chemistry and Physics.* (CRC Press, 1999).
2. Hale, G. M. & Querry, M. R. Optical Constants of Water in the 200-nm to 200-µm Wavelength Region. *Appl. Opt.* **12,** 555-563 (1973).
3. El-Kashef, H. The necessary requirements imposed on polar dielectric laser dye solvents. *Phys. B* **279,** 295-301 (2000).
4. Rheims, J., Köser, J. & Wriedt, T. Refractive-index measurements in the near-IR using an Abbe refractometer. *Meas. Sci. Technol.* **8,** 601-605 (1997).
5. Chu, K.-Y. & Thompson, A. R. Densities and Refractive Indices of Alcohol-Water Solutions of n-Propyl, Isopropyl, and Methyl Alcohols. *J. Chem. Eng. Data* **7,** 358–360 (1962).
6. Moutzouris, K. *et al.* Refractive, dispersive and thermo-optic properties of twelve organic solvents in the visible and near-infrared. *Appl. Phys. B: Lasers Opt.* **116,** 617–622 (2013).
7. *ChemBuddy*. (2011) Available: http://www.refractometer.pl/refraction-datasheet-basic/ (Accessed: 26th May 2014).
8. Butt, H.-J., Graf, K. & Kappl, M. *Physics and chemistry of interfaces.* (Wiley-VCH, 2006).
9. Quéré, D. Rough ideas on wetting. *Physica A* **313,** 32-46 (2002).
